# Risk of Endometrial Cancer and Frequencies of Invasive Endometrial Procedures in Young Breast Cancer Survivors Treated With Tamoxifen: A Nationwide Study

**DOI:** 10.3389/fonc.2021.636378

**Published:** 2021-06-03

**Authors:** Soojeong Choi, Young Jae Lee, Jae Ho Jeong, Jinhong Jung, Jong Won Lee, Hee Jeong Kim, Beom Seok Ko, Byung Ho Son, Sei Hyun Ahn, Yura Lee, Il Yong Chung

**Affiliations:** ^1^ Department of Surgery, Asan Medical Center, University of Ulsan College of Medicine, Seoul, South Korea; ^2^ Department of Obstetrics and Gynecology, Gangneung Asan Hospital, University of Ulsan College of Medicine, Gangneung, South Korea; ^3^ Department of Oncology, Asan Medical Center, University of Ulsan College of Medicine, Seoul, South Korea; ^4^ Department of Radiation Oncology, Asan Medical Center, University of Ulsan College of Medicine, Seoul, South Korea; ^5^ Department of Information Medicine, Asan Medical Center, University of Ulsan College of Medicine, Seoul, South Korea

**Keywords:** breast neoplasms, dilatation and curettage, gynecological examination, tamoxifen, endometrial neoplasms

## Abstract

**Background:**

Although the guidelines recommend gynecological assessment and close monitoring for symptoms of endometrial cancer in postmenopausal breast cancer survivors taking tamoxifen (TAM), the risk of endometrial cancer in young breast cancer survivors has not yet been fully assessed. This study aimed to investigate the risk of developing endometrial cancer and the frequencies of gynecological examinations in young breast cancer survivors taking TAM in South Korea.

**Methods:**

A nationwide retrospective cohort study was conducted using the Health Insurance Review and Assessment Service claims data. Kaplan–Meier analyses and log-rank tests were used to assess the probability of endometrial cancer, benign endometrial conditions, and the probability of invasive endometrial procedure. To analyze the risk of endometrial cancer and benign endometrial conditions, we used a multivariable Cox proportional hazards regression model.

**Results:**

Between 2010 and 2015, 60,545 newly diagnosed female breast cancer survivors were included. The total person–years were 256,099 and 140 (0.23%) patients developed endometrial cancer during the study period. In breast cancer survivors aged ≥60 years [hazard ratio (HR), 5.037; 95% confidence interval (CI), 2.185–11.613], 50–59 years (HR, 4.343; 95% CI, 2.122–8.891), and 40–49 years (HR, 2.121; 95% CI, 1.068–4.213), TAM was associated with an increased risk of endometrial cancer. In subjects aged below 40 years, TAM did not significantly increase the risk of endometrial cancer. However, among the TAM subgroups, breast cancer survivors aged below 40 years [1.61 per 1,000 person–years (PY); HR, 12.460; 95% CI, 2.698–57.522] and aged 40–49 years (2.22 per 1,000 PY; HR, 9.667; 95% CI, 4.966–18.819) with TAM-related endometrial diseases showed significantly increased risks of endometrial cancer. Among the TAM subgroup with benign endometrial conditions, the ratios of the frequency of invasive diagnostic procedures to the incidence of endometrial cancer were higher in subjects under 40 than subjects aged 60 or more.

**Conclusion:**

Young breast cancer survivors with TAM-related benign endometrial diseases are at a higher risk of developing endometrial cancer. Gynecological surveillance should be tailored to the risk of endometrial cancer in young breast cancer survivors to improve the early detection of endometrial cancer and avoid unnecessary invasive procedures.

## Introduction

In hormone receptor-positive breast cancer, use of tamoxifen (TAM) as an adjuvant antihormonal treatment is important (1, 2). TAM is associated with various adverse effects, including hot flashes, vaginal discharge, menstrual irregularities, sexual dysfunction, thromboembolic complications, hyperplasia, and endometrial cancer (3, 4). These adverse effects can reduce patients’ adherence to adjuvant endocrine treatment (5). Endometrial cancer is known as a serious adverse effect related to the use of TAM (6–8).

Most guidelines recommend gynecological assessment for endometrial cancer in postmenopausal breast cancer survivors receiving TAM (9–11). However, routine examinations are not warranted as such surveillance is not effective in enhancing the early detection of endometrial cancer and may lead to more invasive and costly diagnostic procedures in asymptomatic postmenopausal women. Gynecological assessments are recommended to be performed only in postmenopausal women with complaints of abnormal vaginal symptoms (9, 11).

No existing guidelines have recommended gynecological assessment for endometrial cancer in premenopausal women receiving TAM. This is because previous studies did not show a significantly increased risk of endometrial cancer in premenopausal women receiving TAM (1, 10). However, in these studies, the numbers of young breast cancer survivors were small, the incidence of endometrial cancer was low, and assessments for various TAM-related gynecological symptoms were not conducted. Therefore, the risk of endometrial cancer in premenopausal women were possibly underestimated.

Hence, this nationwide retrospective cohort study was conducted using claims data from the Health Insurance Review and Assessment Service (HIRA). This study aimed to investigate the incidence of endometrial cancer in young breast cancer survivors and to analyze the frequency of invasive endometrial procedures in breast cancer survivors who were treated with TAM.

## Materials and Methods

### Data Source and Study Population

Healthcare in South Korea is delivered through a single-payer healthcare system supported by the government. The HIRA has a major role in collecting data of all healthcare services delivered to patients and assessing the healthcare services for reimbursement decisions (12). The HIRA data include patients’ general information, diagnoses, and healthcare services provided such as medications and procedures.

The data registered between January 2008 and December 2018 were extracted from the HIRA database. Patients who were newly diagnosed with breast cancer from January 2010 to December 2015 were included. To exclude prevalent breast cancer patients, we selected a 2-year washout period (from January 2008 to December 2009). Male breast cancer patients, patients with a history of *in situ* carcinoma, patients with presumed metastatic or recurrent breast cancer, and patients who did not undergo breast cancer surgery were excluded. Patients with previous or recent history of other cancer types including endometrial cancer, who underwent hysterectomy, or who had an oophorectomy were also excluded. Moreover, patients without follow-up claims data 1 year after the initiation of endocrine treatment were excluded. Newly diagnosed breast cancer was defined using the C50 code (invasive breast cancer) based on the 10th revision of the International Classification of Diseases (ICD-10) plus the V193 code, which is a claim code for reimbursement of cancer patients (13).

From January 2010 to December 2015, 203,956 breast cancer patients who were assigned with the C50 and V193 codes were identified. We excluded 861 male patients, 6,535 patients with a previous history of *in situ* carcinoma, 13,493 patients with metastatic or recurrent breast cancer, 17,142 patients who did not undergo breast cancer surgery, 16,036 patients with preexisting or who were recently diagnosed with other cancer types, 1,642 patients with previous hysterectomy, 99 patients who previously underwent oophorectomy, and 366 patients who did not have follow-up data 1 year after the initiation of endocrine treatment (**Supplementary Figure 1**).

### Variables and Operational Definitions

Patients’ baseline characteristics and the Charlson Comorbidity Index (CCI) based on ICD-10 codes were analyzed (14). Hypertension (HT), diabetes mellitus (DM), and dyslipidemia were defined using ICD-10 codes (HT, I10–13, 15, and 16; DM, E10–14; and dyslipidemia, E78) and their related medications. Breast cancer treatments were evaluated based on claims data 1 year after the breast cancer diagnosis. Data on surgery, radiation, chemotherapy, endocrine therapy, and trastuzumab were reviewed.

Endometrial cancer was defined using the newly claimed endometrial cancer codes (C55, C54, and D07). Benign endometrial condition was defined using the newly claimed diagnoses such as vaginal bleeding (N93 and N90), endometrial hyperplasia (N851 and N850), and polyp of endometrium (N840). Procedures including endometrial evaluation (aspiration, biopsy, and polypectomy) and dilatation and curettage (D&C) were identified using electronic data interchange codes. In-hospital mortality was assessed.

For landmark analysis, the index date was defined as the date 1 year after the initiation of endocrine therapy or the date of surgery if antihormonal medications were not prescribed.

### Statistical Analysis

The incidence rates of endometrial cancer were compared between the TAM group and no TAM group. In the TAM subgroup stratified according to age at diagnosis (<40, 40–49, 50–59, and ≥60 years), the incidence of endometrial cancer and benign endometrial condition, total frequency of procedures, frequency of procedures per 1,000 person–years, and the ratio of the frequency of invasive diagnostic procedures to the incidence of endometrial cancer were calculated. Kaplan–Meier analysis and the log-rank test were used to assess the disease-free probability of patients with endometrial cancer and benign endometrial condition, and the procedure-free probability of the endometrial evaluation and D&C.

To analyze the risk of endometrial cancer and benign endometrial conditions, we used a Cox proportional hazards regression model adjusted for age at diagnosis, insurance, CCI, previous dyslipidemia, previous diabetes mellitus, previous hypertension, previous polycystic ovarian syndrome, chemotherapy, radiation, and trastuzumab.

Statistical analyses were conducted using R software (version 3.6.1, R Foundation for Statistical Computing, Vienna, Austria) and SAS (version 9.4, SAS Institute Inc., Cary, NC, USA). This study was approved by the Institutional Review Board of Asan Medical Center (IRB no. S2019-1702-0001).

## Results

### Baseline Characteristics

A total of 60,545 breast cancer survivors were included in this analysis. The total person–years were 256,099, and the mean duration after cohort entry was 66 months. Of these patients, 27,034 (44.65%) received TAM, while 33,511 (55.35%) were not treated with TAM (**Table 1**). Of the total patients, 29,635 (48.9%) breast cancer survivors were aged below 50 years, and 7,519 (12.4%) young breast cancer survivors were aged below 40 years (**Supplementary Table 1**). The proportion of breast cancer survivors who received any type of chemotherapy was 60.74% (n = 36,776). During the study period, 68.66% (41,569) of these patients received radiation therapy. Trastuzumab was prescribed in 8,619 (14.23%) patients. All patients underwent breast cancer surgery.

**Table 1:** Baseline characteristics of the study population.

	Tamoxifen		No tamoxifen		Total	
	(n = 27,034, 44.65%)		(n = 33,511, 55.35%)		(n = 60,545, 100%)	
Age at diagnosis (years, mean ± SD)	45.79 ± 8.44		55.39 ± 10.78		51.11 ± 10.90	
Insurance						
Health insurance	26,567	98.27	32,616	97.33	59,183	97.75
Medicare	467	1.73	895	2.67	1,362	2.25
CCI (mean ± SD)	1.59 ± 1.57		2.34 ± 2.04		2.01 ± 1.88	
Previous diabetes mellitus	1,158	4.28	4,018	11.99	5,176	8.55
Previous hypertension	3,804	14.07	11,679	34.85	15,483	25.58
Previous dyslipidemia	3,625	13.40	11,864	35.40	15,489	25.58
Previous PCOS	118	0.44	59	0.18	177	0.29
Chemotherapy	16,045	59.35	20,731	61.86	36,776	60.74
Radiation	19,620	72.58	21,949	65.5	41,569	68.66
Trastuzumab	2,961	10.95	5,658	16.88	8,619	14.23
(Neo)adjuvant endocrine therapy						
None	0		17,521	52.28	17,521	28.94
Tamoxifen	26,374	97.56	0		26,374	43.56
Tamoxifen +AI	660	2.44	0		660	1.09
AI	0		15,990	47.71	15,990	26.41
Endometrial cancer*	98	0.36	42	0.12	140	0.23
Benign endometrial conditions*	7,254	26.83	2,888	8.61	10,142	16.75
In-hospital mortality	465	1.72	1,303	3.89	1,768	2.92
Duration after cohort entry (mean ± SD, month)	66.47 ± 20.34		65.68 ± 20.84		66.03 ± 20.62	

*1 year after the initiation of treatment.

SD, standard deviation; CCI, Charlson Comorbidity Index; PCOS, polycystic ovary syndrome; AI, Aromatase inhibitor.

### Endometrial Cancer in Breast Cancer Survivors

Of the total study population, 140 (0.23%) patients developed endometrial cancer during the study period (**Table 1**). There were 98 (0.36%) and 42 (0.12%) endometrial cancer cases in the TAM group and non-TAM group, respectively.

**Figure 1:**
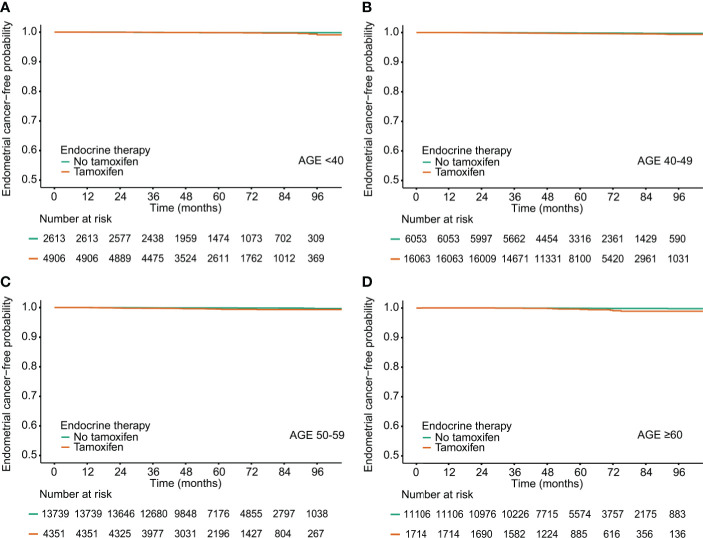
Endometrial cancer-free probability in breast cancer survivors by tamoxifen and age at diagnosis.**(A)** Age<40, **(B)** Age 40-49, **(C)** Age 50-59, **(D)** Age <60.

In breast cancer survivors aged 40 or over, TAM significantly increased the risk of endometrial cancer (**Table 2** and **Figure 1**). The incidence of endometrial cancer in subjects ≥60 years who were treated with TAM was the highest in all age subgroups [1.38 per 1,000 person–years (PY); hazard ratio (HR), 5.037; 95% confidence interval (CI), 2.185–11.613; P<0.001]. However, in subjects aged below 40 years, TAM did not significantly increase the risk of endometrial cancer, and the incidence rate of endometrial cancer was low (0.62 per 1,000 PY; HR, 2.048; 95% CI, 0.658–6.377; P = 0.216).

**Table 2:** Univariate analysis and multivariable Cox regression analysis of endometrial cancer risk related to tamoxifen by age at diagnosis.

Age	Tamoxifen	N	No. of events	Person–years	Incidence rate, per 1,000 person–years	p^a^	Crude HR (95% CI), p				Adjusted HR (95% CI)^b^, p			
<40	No	2,613	4	11,695	0.34		1	(Reference)			1	(Reference)		
	Yes	4,906	13	20,895	0.62	0.300	1.904	0.620	5.851	0.261	2.048	0.658	6.377	0.216
40–49	No	6,053	10	26,577	0.38		1	(Reference)			1	(Reference)		
	Yes	16,063	55	66,919	0.82	0.020	2.187	1.114	4.291	0.023	2.121	1.068	4.213	0.032
50–59	No	13,739	15	58,315	0.26		1	(Reference)			1	(Reference)		
	Yes	4,351	20	18,038	1.11	<0.001	4.308	2.205	8.415	<0.001	4.343	2.122	8.891	<0.001
60≤	No	11,106	13	46,405	0.28		1	(Reference)			1	(Reference)		
	Yes	1,714	10	7,255	1.38	<0.001	4.876	2.138	11.120	<0.001	5.037	2.185	11.613	<0.001

a Log-rank test.

b Adjusted for age at diagnosis (continuous), insurance (health insurance, Medicare), Charlson comorbidity index (continuous), previous hypertension (yes or no), previous diabetes mellitus (yes or no), previous dyslipidemia (yes or no), previous polycystic ovarian syndrome (yes or no), chemotherapy (yes or no), radiation (yes or no), and trastuzumab (yes or no).

CI, confidence interval; HR, hazard ratio.

### Benign Endometrial Conditions in Breast Cancer Survivors

TAM significantly increased the risk of benign endometrial conditions in all age subgroups (**Supplementary Table 2** and **Supplementary Figure 2**). The incidence of benign endometrial conditions in subjects under 40 years who were treated with TAM (88.60 per 1,000 PY) was the highest in all age subgroups. The incidence of benign endometrial conditions in subjects 60 years or more who were not treated with TAM (10.80 per 1,000 PY) was the lowest in all age subgroups.

### Endometrial Cancer in Young Breast Cancer Survivors With Benign Endometrial Conditions

Among the TAM subgroups, benign endometrial conditions were significantly related to an increased risk of endometrial cancer in all age subgroups (**Table 3**). The incidence of endometrial cancer in subjects ≥60 years with benign endometrial conditions was the highest in all age subgroups (5.99 per 1,000 PY). In subjects without benign endometrial conditions, the incidence of endometrial cancer was low.

**Table 3:** Univariate analysis and multivariable Cox regression analysis of endometrial cancer risk related to benign endometrial condition in patients treated with tamoxifen.

Age	Benign endometrial condition	N	No. of events	Person–years	Incidence rate, per 1,000 person–years	p^a^	Crude HR (95% CI)				Adjusted HR (95% CI)^b^			
<40	No	3,396	2	14,049	0.14		1	(Reference)			1	(Reference)			
	Yes	1,510	11	6,846	1.61	<0.001	10.810	2.393	48.830	0.002	12.460	2.698	57.522	0.001	
40–49	No	11,587	11	47,139	0.23		1	(Reference)			1	(Reference)			
	Yes	4,476	44	19,780	2.22	<0.001	9.543	4.927	18.450	<0.001	9.667	4.966	18.819	<0.001	
50–59	No	3,306	5	13,449	0.37		1	(Reference)			1	(Reference)			
	Yes	1,045	15	4,589	3.27	<0.001	8.817	3.203	24.270	<0.001	8.815	3.179	24.444	<0.001	
60≤	No	1,339	0	5,586	0.00		1	(Reference)			1	(Reference)			
	Yes	375	10	1,669	5.99	<0.001	NA				NA			

a Log-rank test.

b Adjusted for age at diagnosis (continuous), insurance (health insurance, Medicare), Charlson comorbidity index (continuous), previous hypertension (yes or no), previous diabetes mellitus (yes or no), previous dyslipidemia (yes or no), previous polycystic ovarian syndrome (yes or no), chemotherapy (yes or no), radiation (yes or no), and trastuzumab (yes or no).

CI, confidence interval; HR, hazard ratio.

In younger breast cancer survivors aged under 40 years (HR, 12.460; 95% CI, 2.698–57.522; *P* = 0.001) and 40–49 years (HR, 9.667; 95% CI, 4.966–18.819; *P* < 0.001), benign endometrial conditions significantly increased the risk of endometrial cancer, although the actual incidence rates of endometrial cancer were lower than those of their older counterparts (1.61 per 1,000 PY in subjects under 40 years; 2.22 per 1,000 PY in subjects aged 40–49 years).

### Frequencies of Invasive Endometrial Procedures in Breast Cancer Survivors

**Figure 2:**
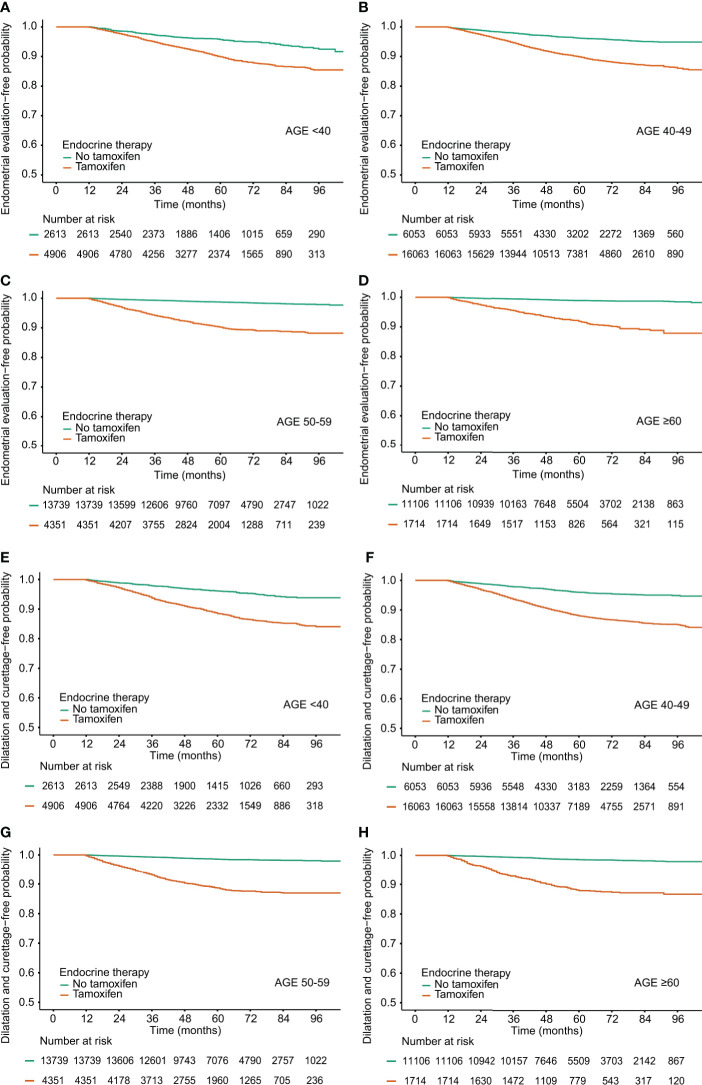
Procedure-free probability in breast cancer survivors by tamoxifen and age at diagnosis. **(A–D)** Endometrial evaluation, **(E–H)** Dilatation and Curettage.

The frequencies of invasive endometrial procedures were higher in the TAM groups than those in the non-TAM subgroups (**Table 4** and **Figure 2**). Among the TAM group aged 60 years or more, invasive endometrial evaluations and D&C were performed about 18 and 23 times to detect one endometrial cancer, respectively. However, in the TAM group aged below 40 years, invasive endometrial evaluations and D&C were conducted more than 46 and 54 times to find one endometrial cancer, respectively.

**Table 4:** Frequencies of endometrial procedures in breast cancer survivors 1 year after initiation of treatment.

	N	Endometrial evaluation					Dilatation and curettage					
		Patients, no. (%)		Procedures, no.	Procedure rate (per 1,000 person–years)	Ratio^a^	Patients, no. (%)		Procedures, no.	Procedure rate (per 1,000 person–years)	Ratio^b^
Tamoxifen											
<40	4,906	492	10.0	606	29.0	46.6	557	11.4	707	33.8	54.4
40–49	16,063	1,592	9.91	2,021	30.2	36.7	1,799	11.2	2,269	33.9	41.3
50–59	4,351	399	9.17	505	28.0	25.2	465	10.7	581	32.2	29.0
60≤	1,714	144	8.40	179	24.7	17.9	184	10.7	234	32.0	23.4
No tamoxifen											
<40	2,613	128	4.90	154	13.20	38.50	111	4.25	141	12.10	35.20
40–49	6,053	230	3.80	290	10.90	29.00	232	3.83	279	10.50	27.90
50–59	13,739	187	1.36	215	3.69	14.30	190	1.38	218	3.74	14.50
60≤	11,106	124	1.12	153	3.30	11.80	157	1.41	178	3.84	13.70

a Ratio = the rate of endometrial evaluation/the rate of endometrial cancer.

b Ratio = the rate of dilation and curettage/the rate of endometrial cancer.

Among breast cancer survivors treated with TAM, the rates of invasive endometrial procedures were higher in subjects with benign endometrial conditions (**Supplementary Table 3**). Among the TAM-treated subjects with benign endometrial conditions, the ratios of the frequency of invasive diagnostic procedures to the incidence of endometrial cancer were 49.0 (endometrial evaluation) and 59.5 (D&C) in subjects under 40 years. However, in subjects aged 60 or more, the ratios were 14.6 (endometrial evaluation) and 21.2 (D&C), respectively.

## Discussion

This study demonstrated that the risk of endometrial cancer increased in young breast cancer survivors on TAM. Even young breast cancer survivors aged below 40 years who had TAM-related benign endometrial conditions such as vaginal bleeding, endometrial hyperplasia, and endometrial polyp showed a significantly increased risk of endometrial cancer compared with those without benign endometrial conditions. Although the incidence rates of endometrial cancer in younger breast cancer survivors were lower than those of their older counterparts; the frequencies of invasive endometrial procedures were higher in younger breast cancer survivors than in their older counterparts.

To our knowledge, this is the first nationwide study to demonstrate the increased risk of endometrial cancer in young breast cancer survivors treated with TAM. Previous studies were limited by the fact that the sample size was not enough to assess the endometrial cancer risk in premenopausal breast cancer survivors on TAM treatment. The National Surgical Adjuvant Breast and Bowel Project (NSABP) P-1 study, where the current guidelines are based, indicated that treatment with TAM did not increase the endometrial cancer risk in premenopausal women and that additional monitoring was not required (10). However, this study enrolled 5,077 patients aged below 50 years (2,596 in the placebo group and 2,581 in the TAM group), and the number of participants aged below 40 years was 244 (185 in the placebo group and 159 in the TAM group). Of the total sample aged below 50 years, 17 (eight in the placebo group and nine in the TAM group) patients developed endometrial cancer. Treatment with TAM did not significantly increase the risk of endometrial cancer in women aged below 50 years (risk ratio, 1.21; 95% CI, 0.41–3.60). From the NASBP B-14 study, the incidence of endometrial cancer in TAM-treated breast cancer survivors were investigated (1). This study enrolled 2,843 participations (1,424 in the placebo group and 1,419 in the TAM group), and only 62 patients were aged below 50 years. A total of 15 patients (zero in the placebo group and 15 in the TAM group) developed endometrial cancer; of them, only one patient in the TAM group was aged below 50 years. These results suggest that the previous studies may underestimate the risk of endometrial cancer in TAM-treated premenopausal breast cancer patients. In our study, 29,635 breast cancer survivors aged below 50 years were included, and 68 of them developed endometrial cancer. Meanwhile, 7,519 young breast cancer survivors aged below 40 years were investigated.

Because the current international guidelines are based on the previous studies, gynecological assessment is not recommended for premenopausal women taking TAM. According to the American Cancer Society/American Society of Clinical Oncology Survivorship Care Guidelines, clinicians should perform annual gynecological assessments in postmenopausal women taking TAM, and patients should inform their physicians if unexpected bleeding occurs (11). According to the 2014 American College of Obstetricians and Gynecologists guidelines, postmenopausal women taking TAM should monitor for symptoms of endometrial disease or cancer (9). In the European Society for Medical Oncology guidelines for postmenopausal breast cancer survivors, appropriate diagnostic tests are recommended to be carried out in those with symptoms of endometrial hyperplasia (15).

In our study, young breast cancer survivors aged below 50 years who had TAM-related endometrial diseases or symptoms showed a significantly increased risk of endometrial cancer. This suggests that gynecological assessments of endometrial cancer in these young breast cancer survivors on higher risk of developing endometrial cancer should be considered. However, the actual incidence rates of endometrial cancer in younger breast cancer survivors (1.61 per 1,000 PY in subjects under 40 years; 2.22 per 1,000 PY in subjects aged 40–49 years) were lower than those of their older counterparts (3.27 per 1,000 PY in subjects in their 50s; 5.99 per 1,000 PY in subjects ≥60 years), and therefore balanced decisions on screening for endometrial cancer should be made.

The guidelines indicated that more attention should be paid to the symptoms related to endometrial hyperplasia. In patients who did not develop any adverse effects after taking TAM, initial screening and regular Papanicolaou smear examinations are usually recommended because the benefits of routine endometrial surveillance in asymptomatic patients on TAM therapy remain unknown. There are no clear guidelines on which gynecological examination should be performed (16, 17). In previous studies, endometrial biopsy or transvaginal ultrasound was not used to screen asymptomatic women receiving TAM (18, 19). Although another previous study reported that there was no significant increase in the incidence of endometrial cancer in young breast cancer patients aged below 40 years, the study did not analyze the presence or absence of benign endometrial symptoms and disease (20). In our study, the risk of endometrial cancer increased only in young breast cancer survivors with TAM-related endometrial benign disease. Hence, it may be appropriate to assess for signs of endometrial hyperplasia like abnormal bleeding, including spotting and abnormal vaginal discharge in young breast cancer survivors on TAM.

In a large-scale randomized controlled trial of female breast cancer survivors, treatment with TAM for 10 years further reduced the recurrence and mortality rates, particularly after 10 years, compared with discontinuing this treatment after 5 years (21, 22). Extended adjuvant TAM therapy is associated with an increase in the risk of endometrial cancer (23). The longer the TAM treatment period is, the more important endometrial cancer screening through gynecological examination can become. Young breast cancer survivors undergoing extended endocrine therapy with TAM up to 10 years should be informed of the possible risk of developing endometrial cancer and to monitor for symptoms related to benign endometrial disease. TAM administered at dose of 5 mg/d for 3 years can reduce the recurrence of breast cancer by 50% with a limited toxicity and may be used as a new treatment option for these patients (24).

To the best of our knowledge, the frequencies of gynecological diagnostic procedures among young breast cancer survivors in the real-world setting have rarely been reported (25). Invasive procedure can cause harm to these young patients. In this study, young breast cancer survivors with TAM-related endometrial conditions showed a significantly increased risk of endometrial cancer. However, the ratios of endometrial evaluation and D&C to the incidence of endometrial cancer in breast cancer survivors aged below 50 years were higher than those in breast cancer survivors aged 50 years and older. Considering the actual incidence of endometrial cancer by age, these invasive procedures should be cautiously opted to avoid causing unnecessary harm.

This study has some limitations. First, the HIRA data lacked information on laboratory examinations, imaging studies, family history, and pathological outcomes such as cancer stage, hormone receptor status, and HER2 overexpression. Second, patients’ adherence to individual treatments could not be specified. Third, vaginal ultrasound exams were not able to be analyzed because information about ultrasound is not archived in the HIRA database. Fourth, clinical results such as recurrence, metastasis, or the cause of death were not available. Only in-hospital mortality was assessed. Lastly, although pregnancy is known to reduce the risk of endometrial cancer, the multivariate analyses in this study were not adjusted by this factor due to insufficient data.

In conclusion, young breast cancer survivors with TAM-related endometrial benign disease are at a higher risk of developing endometrial cancer. Clinicians should assess for benign endometrial conditions in premenopausal breast cancer survivors who are taking TAM. Gynecological surveillance should be tailored to the risk of endometrial cancer in young breast cancer survivors to improve the early detection of endometrial cancer and avoid unnecessary invasive procedures.

## Data Availability Statement

The datasets generated for this study is not publicly available. These datasets are from the Korean national database, and are not allowed to be extracted from the server by laws. Requests to access the datasets should be directed to the Health Insurance Review and Assessment Service.

## Ethics Statement

The studies involving human participants were reviewed and approved by the Institutional Review Board of Asan Medical Center (IRB no. S2019-1702-0001). Written informed consent from the participants’ legal guardian/next of kin was not required in accordance with the national legislation and the institutional requirements.

## Author Contributions

SC: planned, wrote, and revised the article. JHJ, YJL, JJ, JL, HK, BK, BS, and SA reviewed and edited the article. YL: planned, analyzed the data, and performed the statistics. IC: planned, wrote, revised, supervised, and designed the concept of the article. All authors contributed to the article and approved the submitted version.

## Conflict of Interest

The authors declare that the research was conducted in the absence of any commercial or financial relationships that could be construed as a potential conflict of interest.

## Supplementary Material

Supplementary Figure 1 Study population.

Supplementary Figure 2 Benign endometrial condition-free probability in breast cancer survivors by tamoxifen and age at diagnosis.
