# Supplementary figures and images for: Risk of Endometrial Cancer and Frequencies of Invasive Endometrial Procedures in Young Breast Cancer Survivors Treated With Tamoxifen: A Nationwide Study
Source: Front Oncol. 2021 Jun 3;11:636378. doi: 10.3389/fonc.2021.636378 (PMC8209428; doi:10.3389/fonc.2021.636378)

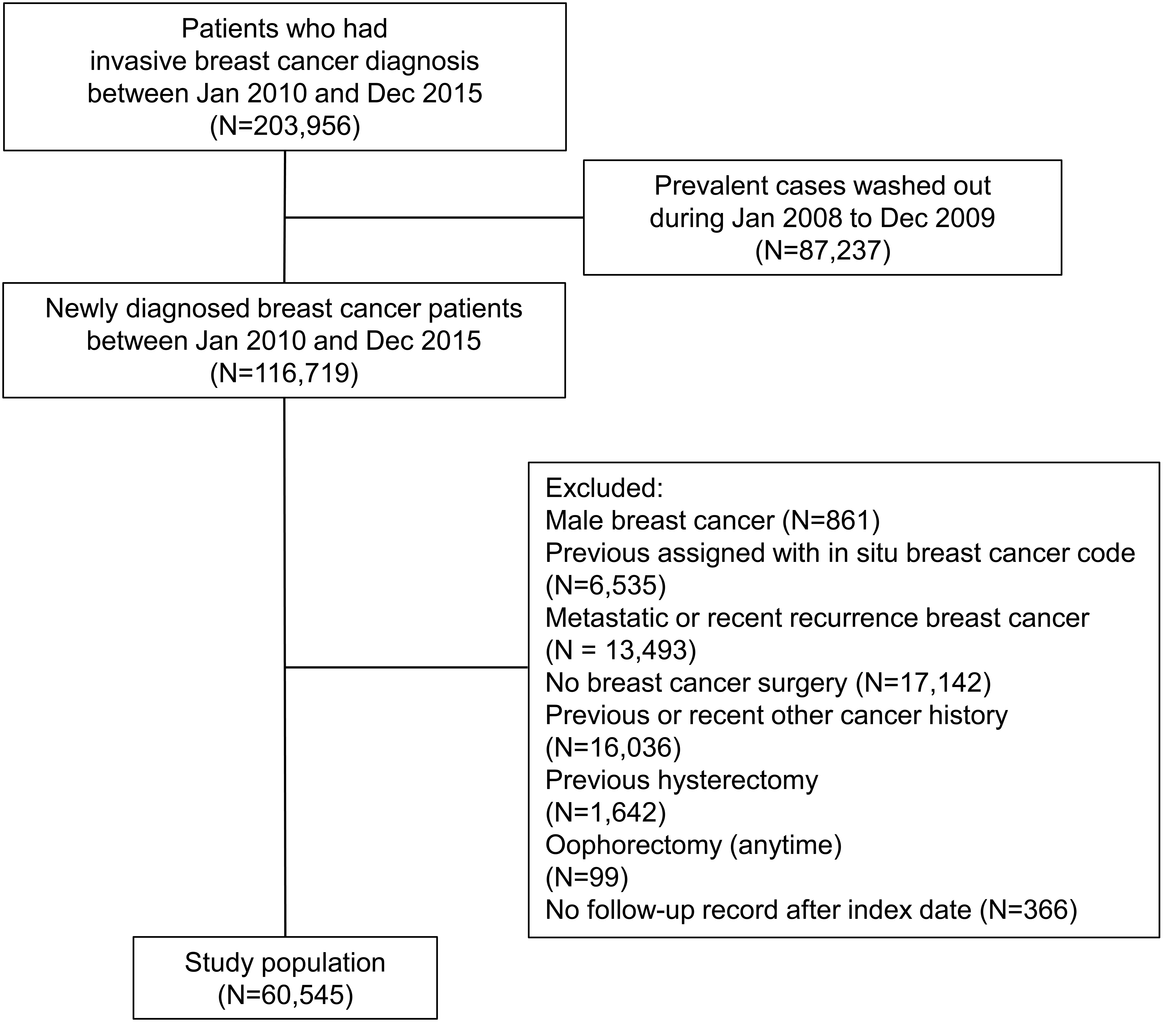

Supplement: Supplementary Figure 1 — Study population. [file Image_1.tif]

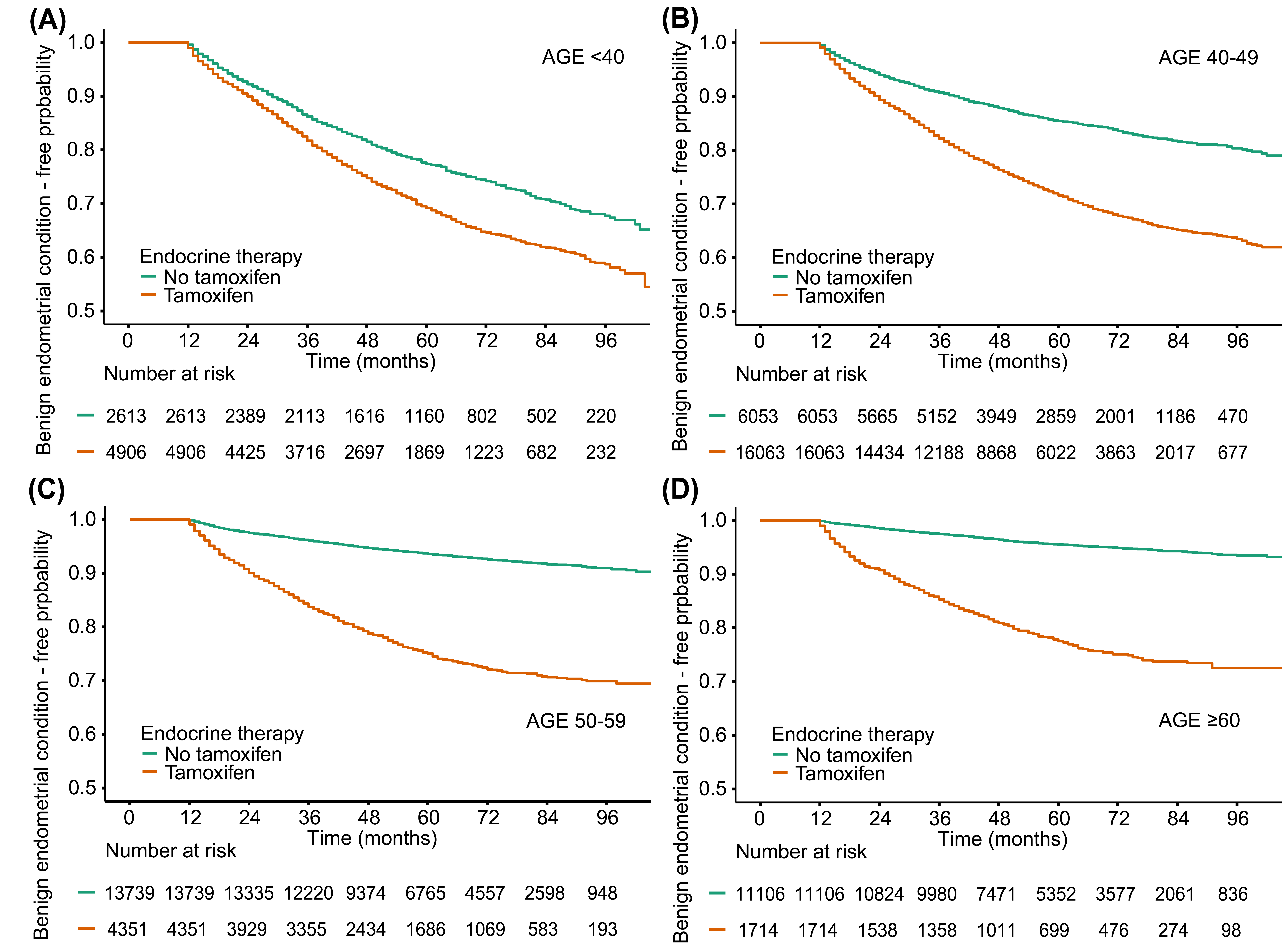

Supplement: Supplementary Figure 2 — Benign endometrial condition-free probability in breast cancer survivors by tamoxifen and age at diagnosis. [file Image_2.tif]
