# Supplementary material for: Risk of Endometrial Cancer and Frequencies of Invasive Endometrial Procedures in Young Breast Cancer Survivors Treated With Tamoxifen: A Nationwide Study
Source: Front Oncol. 2021 Jun 3;11:636378. doi: 10.3389/fonc.2021.636378 (PMC8209428; doi:10.3389/fonc.2021.636378)
Supplement: Supplementary file 3 [file Table_1.docx]

| Supplementary Table 1. Characteristics of subjects by age at diagnosis | | | | |
| --- | --- | --- | --- | --- |
| Age <40 |  |  |  |  |
|  | Tamoxifen | | No tamoxifen | |
|  | (n =4906, 65.25%) | | (n =2613, 34.75%) | |
| Age at diagnosis (years, Mean ± SD) | 35.36±3.58 | | 34.52±3.88 | |
| Insurance |  |  |  |  |
| Health insurance | 4865 | 99.16 | 2592 | 99.20 |
| Medicare | 41 | 0.84 | 21 | 0.80 |
| CCI (Mean ± SD) | 1.23±1.18 | | 1.20±1.16 | |
| previous diabetes mellitus | 39 | 0.79 | 20 | 0.77 |
| previous hypertension | 166 | 3.38 | 66 | 2.53 |
| previous dyslipidemia | 148 | 3.02 | 89 | 3.41 |
| previous PCOS | 64 | 1.30 | 32 | 1.22 |
| Chemotherapy | 3361 | 68.51 | 1888 | 72.25 |
| Radiation | 3570 | 72.77 | 1730 | 66.21 |
| Trastuzumab | 699 | 14.25 | 388 | 14.85 |
| (Neo)adjuvant endocrine therapy |  |  |  |  |
| None | 0 | 0.00 | 2605 | 99.69 |
| Tamoxifen | 4895 | 99.78 | 0 |  |
| Tamoxifen+ AI | 11 | 0.22 | 0 |  |
| AI | 0 | 0.00 | 8 | 0.31 |
| Endometrial cancer* | 13 | 0.26 | 4 | 0.15 |
| Benign endometrial conditions* | 1510 | 30.78 | 618 | 23.65 |
| In-hospital mortality | 96 | 1.96 | 124 | 4.75 |
| Duration after cohort entry  (mean±SD, month) | 67.85±20.49 | | 67.12±21.50 | |
|  |  |  |  |  |

| Age 40-49 |  |  |  |  |
| --- | --- | --- | --- | --- |
|  | Tamoxifen | | No tamoxifen | |
|  | (n =16063, 72.63%) | | (n =6053, 27.37%) | |
| Age at diagnosis (years, Mean ± SD) | 44.75±2.75 | | 45.18±2.88 | |
| Insurance |  |  |  |  |
| Health insurance | 15802 | 98.38 | 5913 | 97.69 |
| Medicare | 261 | 1.62 | 140 | 2.31 |
| CCI (Mean ± SD) | 1.40±1.35 | | 1.44±1.42 | |
| previous diabetes mellitus | 436 | 2.71 | 179 | 2.96 |
| previous hypertension | 1532 | 9.54 | 663 | 10.95 |
| previous dyslipidemia | 1513 | 9.42 | 758 | 12.52 |
| previous PCOS | 50 | 0.31 | 18 | 0.30 |
| Chemotherapy | 9715 | 60.48 | 4179 | 69.04 |
| Radiation | 11987 | 74.62 | 3809 | 62.93 |
| Trastuzumab | 1704 | 10.61 | 1108 | 18.30 |
| (Neo)adjuvant endocrine therapy |  |  |  |  |
| None | 0 | 0.00 | 5270 | 87.06 |
| Tamoxifen | 15956 | 99.33 | 0 |  |
| Tamoxifen+ AI | 107 | 0.67 | 0 |  |
| AI | 0 | 0.00 | 783 | 12.94 |
| Endometrial cancer* | 55 | 0.34 | 10 | 0.17 |
| Benign endometrial conditions* | 4476 | 27.87 | 899 | 14.85 |
| In-hospital mortality | 210 | 1.31 | 208 | 3.44 |
| Duration after cohort entry  (mean±SD, month) | 66.31±20.23 | | 66.34±21.16 | |
|  |  |  |  |  |

| Age 50-59 |  |  |  |  |
| --- | --- | --- | --- | --- |
|  | Tamoxifen | | No tamoxifen | |
|  | (n =4351, 24.05%) | | (n =13739, 75.95%) | |
| Age at diagnosis (years, Mean ± SD) | 52.37±2.54 | | 54.37±2.75 | |
| Insurance |  |  |  |  |
| Health insurance | 4260 | 97.91 | 13487 | 98.17 |
| Medicare | 91 | 2.09 | 252 | 1.83 |
| CCI (Mean ± SD) | 1.95±1.68 | | 2.16±1.83 | |
| previous diabetes mellitus | 261 | 6.00 | 1192 | 8.68 |
| previous hypertension | 999 | 22.96 | 4068 | 29.61 |
| previous dyslipidemia | 1016 | 23.35 | 4745 | 34.54 |
| previous PCOS | 4 | 0.09 | 9 | 0.07 |
| Chemotherapy | 2383 | 54.77 | 9242 | 67.27 |
| Radiation | 3162 | 72.67 | 9864 | 71.80 |
| Trastuzumab | 454 | 10.43 | 2787 | 20.29 |
| (Neo)adjuvant endocrine therapy |  |  |  |  |
| None | 0 | 0.00 | 5803 | 42.24 |
| Tamoxifen | 4061 | 93.33 | 0 |  |
| Tamoxifen+ AI | 290 | 6.67 | 0 |  |
| AI | 0 | 0.00 | 7936 | 57.76 |
| Endometrial cancer* | 20 | 0.46 | 15 | 0.11 |
| Benign endometrial conditions* | 1045 | 24.02 | 891 | 6.49 |
| In-hospital mortality | 70 | 1.61 | 376 | 2.74 |
| Duration after cohort entry  (mean±SD, month) | 65.79±20.27 | | 65.97±20.47 | |
|  |  |  |  |  |

| Age≥60 |  |  |  |  |
| --- | --- | --- | --- | --- |
|  | Tamoxifen | | No tamoxifen | |
|  | (n =1714, 13.37%) | | (n =11106, 86.63%) | |
| Age at diagnosis (years, Mean ± SD) | 68.69±6.59 | | 67.14±5.86 | |
| Insurance |  |  |  |  |
| Health insurance | 1640 | 95.68 | 10624 | 95.66 |
| Medicare | 74 | 4.32 | 482 | 4.34 |
| CCI (Mean ± SD) | 3.46±2.39 | | 3.32±2.31 | |
| previous diabetes mellitus | 422 | 24.62 | 2627 | 23.65 |
| previous hypertension | 1107 | 64.59 | 6882 | 61.97 |
| previous dyslipidemia | 948 | 55.31 | 6272 | 56.47 |
| previous PCOS | 0 | 0.00 | 0 | 0.00 |
| Chemotherapy | 586 | 34.19 | 5422 | 48.82 |
| Radiation | 901 | 52.57 | 6546 | 58.94 |
| Trastuzumab | 104 | 6.67 | 1375 | 12.38 |
| (Neo)adjuvant endocrine therapy |  |  |  |  |
| None | 0 | 0.00 | 3843 | 34.60 |
| Tamoxifen | 1462 | 85.30 | 0 |  |
| Tamoxifen+ AI | 252 | 14.70 | 0 |  |
| AI | 0 | 0.00 | 1263 | 65.40 |
| Endometrial cancer* | 10 | 0.58 | 13 | 0.12 |
| Benign endometrial conditions* | 375 | 21.88 | 490 | 4.41 |
| In-hospital mortality | 89 | 5.19 | 595 | 5.36 |
| Duration after cohort entry  (mean±SD, month) | 65.76±21.05 | | 64.63±20.91 | |
| * 1 year after the start of treatment  **SD**: standard deviation **CCI**: Charlson Comorbidity index **PCOS**: polycystic ovary syndrome, **AI**: Aromatase inhibitor | | | | |

| Supplementary Table 2. Univariate analysis and multivariable Cox regression analysis of endometrial benign disease risk related to tamoxifen by age at diagnosis | | | | | | | | | | | | | | |
| --- | --- | --- | --- | --- | --- | --- | --- | --- | --- | --- | --- | --- | --- | --- |
| Age at diagnosis | Tamoxifen | N | No. of events | Person-years | Incidence rate,  per 1000  person-years | p^a^ | Crude HR (95% CI) | | | | Adjusted HR (95% CI) ^b^ | | | |
| <40 | No | 2,613 | 618 | 9,952 | 62.10 |  | 1 | (Reference) | |  | 1 | (Reference) | |  |
|  | Yes | 4,906 | 1,510 | 17,040 | 88.60 | <0.001 | 1.406 | 1.280 | 1.544 | <0.001 | 1.379 | 1.254 | 1.516 | <0.001 |
| 40–49 | No | 6,053 | 899 | 24,008 | 37.40 |  | 1 | (Reference) | |  | 1 | (Reference) | |  |
|  | Yes | 16,063 | 4,476 | 55,308 | 80.90 | <0.001 | 2.089 | 1.945 | 2.245 | <0.001 | 1.985 | 1.846 | 2.136 | <0.001 |
| 50–59 | No | 13,739 | 891 | 56,020 | 15.90 |  | 1 | (Reference) | |  | 1 | (Reference) | |  |
|  | Yes | 4,351 | 1,045 | 15,210 | 68.70 | <0.001 | 4.182 | 3.824 | 4.574 | <0.001 | 3.809 | 3.457 | 4.196 | <0.001 |
| 60≤ | No | 11,106 | 490 | 45,187 | 10.80 |  | 1 | (Reference) | |  | 1 | (Reference) | |  |
|  | Yes | 1,714 | 375 | 6,201 | 60.50 | <0.001 | 5.486 | 4.795 | 6.275 | <0.001 | 5.775 | 5.036 | 6.622 | <0.001 |
| ^a^Log-rank test | |  |  |  |  |  |  |  |  |  |  |  |  |  |
| ^b^Adjusted for age at diagnosis (continuous), insurance (health insurance, Medicare), Charlson comorbidity index (continuous), previous hypertension (yes or no), previous diabetes mellitus (yes or no), previous dyslipidemia (yes or no), previous polycystic ovarian syndrome (yes or no), chemotherapy (yes or no), radiation (yes or no), and trastuzumab (yes or no)  **CI**: confidence interval, **HR**: hazard ratio | | | | | | | | | | | | | | |

| Supplementary Table 3. Frequencies of endometrial procedures in breast cancer survivors 1 year after initiation of tamoxifen by benign endometrial condition | | | | | | | | | | | | |
| --- | --- | --- | --- | --- | --- | --- | --- | --- | --- | --- | --- | --- |
|  |  |  |  |  |  |  |  |  |  |  | |  |
|  |  | Endometrial evaluation | | | | | Dilatation and curettage | | | | | |
|  | N | Patients,  no. (%) | | Procedures, no. | Procedure rate (per 1000 person-years) | Ratio^a^ | Patients,  no. (%) | | Procedures, no. | Procedure rate (per 1,000 person-years) | | Ratio^b^ |
| Benign endometrial condition | | | |  |  |  |  |  |  |  | |  |
| <40 | 1,510 | 429 | 28.40 | 539 | 78.70 | 49 | 506 | 33.50 | 655 | 95.70 | | 59.50 |
| 40–49 | 4,476 | 1,305 | 29.20 | 1,689 | 85.40 | 38.40 | 1,643 | 36.70 | 2,095 | 1060 | | 47.60 |
| 50–59 | 1,045 | 331 | 31.70 | 427 | 93 | 28.50 | 415 | 39.70 | 528 | 115 | | 35.20 |
| 60≤ | 375 | 117 | 31.20 | 146 | 87.50 | 14.60 | 165 | 44 | 212 | 127 | | 21.20 |
| No benign endometrial condition | | | | |  |  |  |  |  |  | |  |
| <40 | 3,396 | 63 | 1.86 | 67 | 4.77 | 33.50 | 51 | 1.50 | 52 | 3.70 | | 26 |
| 40–49 | 11,587 | 287 | 2.48 | 332 | 7.04 | 30.20 | 156 | 1.35 | 174 | 3.69 | | 15.80 |
| 50–59 | 3,306 | 68 | 2.06 | 78 | 5.80 | 15.60 | 50 | 1.51 | 53 | 3.94 | | 10.60 |
| 60≤ | 1,339 | 27 | 2.02 | 33 | 5.91 | NA | 19 | 1.42 | 22 | 3.94 | | NA |
| ^a^Ratio = the rate of endometrial evaluation/the rate of endometrial cancer | | | | | | | | | |  |  | |
| ^b^Ratio = the rate of dilation and curettage/the rate of endometrial cancer | | | | | | | | | |  |  | |
